# Crystal structure of (*Z*)-4-methylbenzyl 3-[1-(5-methylpyridin-2-yl)ethylidene]dithiocarbazate^1^

**DOI:** 10.1107/S205698901502407X

**Published:** 2015-12-19

**Authors:** Thahira Begum S. A. Ravoof, Edward R. T. Tiekink, Siti Aminah Omar, Sharifa Zaithun Begum, Mohamed I. M. Tahir

**Affiliations:** a Department of Chemistry, Universiti Putra Malaysia, 43400 Serdang, Malaysia; b Centre for Crystalline Materials, Faculty of Science and Technology, Sunway University, 47500 Bandar Sunway, Selangor Darul Ehsan, Malaysia

**Keywords:** crystal structure, hydrogen bonding, di­thio­carbazate

## Abstract

In the title di­thio­carbazate compound, C_17_H_19_N_3_S_2_, the central CN_2_S_2_ residue is essentially planar (r.m.s. deviation = 0.0288 Å) and forms dihedral angles of 9.77 (8) and 77.47 (7)° with the substituted-pyridyl and *p*-tolyl rings, respectively, indicating a highly twisted mol­ecule; the dihedral angle between the rings is 85.56 (8)°. The configuration about the C=N bond is *Z*, which allows for the formation of an intra­molecular N—H⋯N(pyrid­yl) hydrogen bond. The packing features tolyl-methyl-C—H⋯N(imine), pyridyl-C—H⋯π(tol­yl) and π–π inter­actions [between pyridyl rings with a distance = 3.7946 (13) Å], which generates jagged supra­molecular layers that stack along the *b* axis with no directional inter­actions between them.

## Related literature   

For the structure of the 4-methyl­pyridin-2-yl derivative, with an *E* configuration for the C=N bond, allowing for the formation of centrosymmetric {⋯HNCS}_2_ synthons in the crystal, see: Omar *et al.* (2014 ▸). For the synthesis, see: Ravoof *et al.* (2010 ▸).
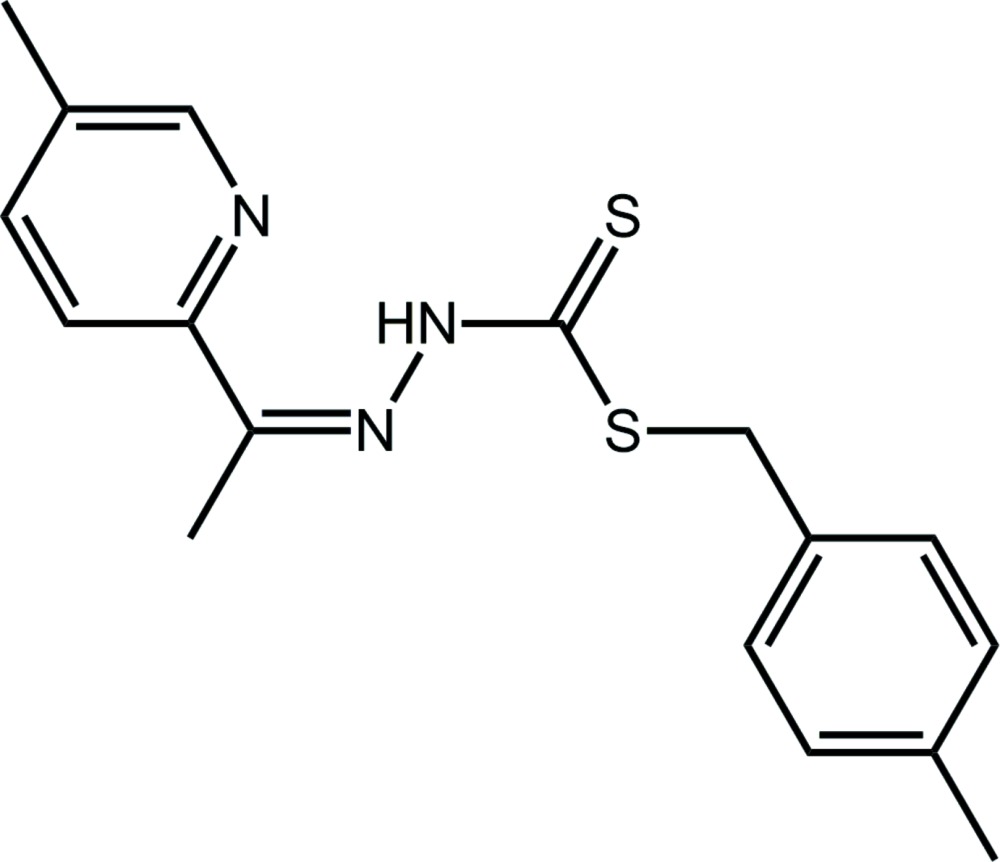


## Experimental   

### Crystal data  

C_17_H_19_N_3_S_2_
*M* *_r_* = 329.47
Monoclinic, 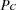

*a* = 9.0073 (2) Å
*b* = 12.3856 (2) Å
*c* = 7.5553 (1) Å
β = 98.620 (2)°
*V* = 833.35 (3) Å^3^
*Z* = 2
Cu *K*α radiation
μ = 2.88 mm^−1^
*T* = 100 K
0.31 × 0.22 × 0.18 mm

### Data collection  

Agilent Xcalibur, Eos, Gemini diffractometer
Absorption correction: multi-scan (*CrysAlis PRO*; Agilent, 2011 ▸) *T*_min_ = 0.43, *T*_max_ = 0.60
16118 measured reflections
3195 independent reflections
3191 reflections with *I* > 2σ(*I*)
*R*_int_ = 0.021

### Refinement  

*R*[*F* ^2^ > 2σ(*F* ^2^)] = 0.030
*wR*(*F* ^2^) = 0.081
*S* = 1.05
3195 reflections
205 parameters
3 restraints
H-atom parameters constrained
Δρ_max_ = 0.28 e Å^−3^
Δρ_min_ = −0.30 e Å^−3^
Absolute structure: Flack *x* determined using 1558 quotients [(*I* ^+^)−(*I* ^−^)]/[(*I* ^+^)+(*I* ^−^)] (Parsons *et al.*, 2013 ▸).
Absolute structure parameter: −0.011 (13)

### 

Data collection: *CrysAlis PRO* (Agilent, 2011 ▸); cell refinement: *CrysAlis PRO*; data reduction: *CrysAlis PRO*; program(s) used to solve structure: *SHELXS97* (Sheldrick, 2008 ▸); program(s) used to refine structure: *SHELXL2014*/7 (Sheldrick, 2015 ▸); molecular graphics: *ORTEP-3 for Windows* (Farrugia, 2012 ▸) and *DIAMOND* (Brandenburg, 2006 ▸); software used to prepare material for publication: *publCIF* (Westrip, 2010 ▸).

## Supplementary Material

Crystal structure: contains datablock(s) 1, I. DOI: 10.1107/S205698901502407X/hb7554sup1.cif

Structure factors: contains datablock(s) I. DOI: 10.1107/S205698901502407X/hb7554Isup2.hkl

**Fig. 1.**
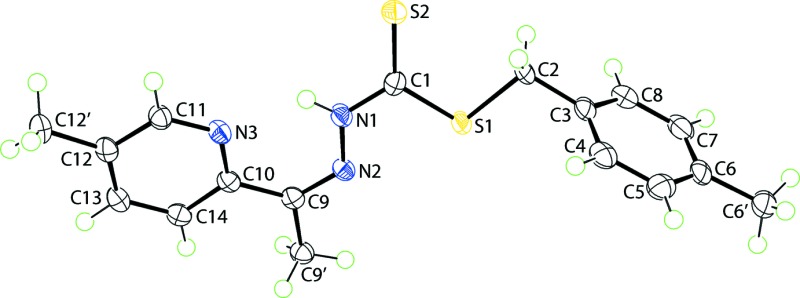
The molecular structure of the title compound showing the atom-labelling scheme and displacement ellipsoids at the 70% probability level.

**Fig. 2.**
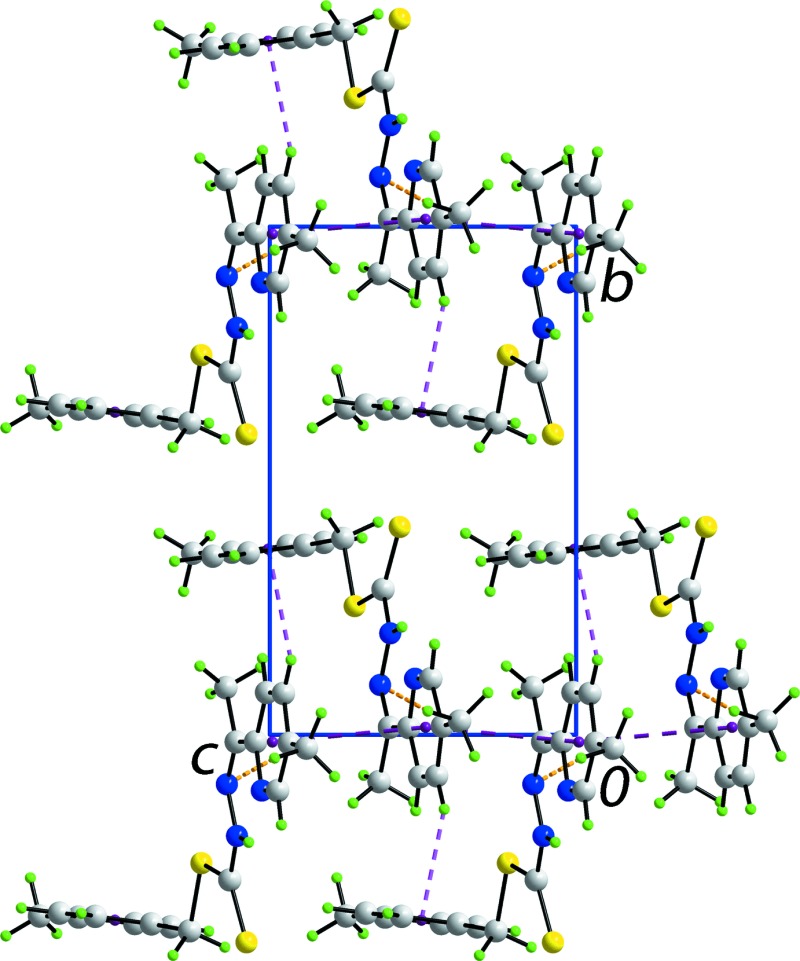
A view of the unit-cell contents in projection down the a axis. The tolyl-methyl-C—H···N(imine), pyridyl-C—H···π(tolyl) and π—π interactions are shown as orange, pink and orange dashed lines, respectively.

. DOI: 10.1107/S205698901502407X/hb7554fig1.tif
The mol­ecular structure of the title compound showing the atom-labelling scheme and displacement ellipsoids at the 70% probability level.

a . DOI: 10.1107/S205698901502407X/hb7554fig2.tif
A view of the unit-cell contents in projection down the *a* axis. The tolyl-methyl-C—H⋯N(imine), pyridyl-C—H⋯π(tol­yl) and π—π inter­actions are shown as orange, pink and orange dashed lines, respectively.

CCDC reference: 1442456

Additional supporting information:  crystallographic information; 3D view; checkCIF report

## Figures and Tables

**Table 1:** Hydrogen-bond geometry (Å, °) *Cg*1 is the centroid of the C3–C8 ring.

*D*—H⋯*A*	*D*—H	H⋯*A*	*D*⋯*A*	*D*—H⋯*A*
N1—H1*N*⋯N3	0.88 (2)	1.98 (3)	2.624 (3)	130 (3)
C12′—H12*C*⋯N2^i^	0.98	2.58	3.483 (3)	154
C13—H13⋯*Cg*1^ii^	0.95	2.76	3.582 (3)	145

Symmetry codes: (i) 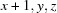; (ii) 
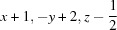
.

## supplementary crystallographic information

### S2. Experimental

The precursor molecule, *S*-4-methylbenzyldithiocarbazate, was prepared by adapting the literature procedure of Ravoof *et al.* (2010). Thus, KOH (11.2 g, 0.2 mol) was dissolved in absolute ethanol (70 ml) and to this solution was added hydrazine hydrate (10 g, 0.2 mol) followed by cooling in an ice-salt bath. Drop wise addition of carbon disulfide (15.2 g, 0.2 mol) with constant stirring over 1 h followed. The two layers that subsequently formed were separated. The light-brown lower layer was dissolved in 40% ethanol (60 ml) below 268 K. The mixture was kept in an ice-bath and 4-methylbenzyl chloride (26.5 ml, 0.2 mol) was added drop wise with vigorous stirring. The major product, which was white and sticky was filtered and left overnight to dry over anhydrous silica gel in a desiccator. Recrystallization to yield analytically pure *S*-4-methylbenzyldithiocarbazate was achieved from hot acetonitrile. Yield: 82%; *M*.pt: 160–161 °*C*.

*S*-4-Methylbenzyldithiocarbazate (2.12 g, 0.01 mol) was dissolved in hot acetonitrile (100 ml) and added to an equimolar solution of 5-methyl-pyridine-2-aldehyde (1.21 g, 0.01 mol) in ethanol (25 ml). The mixture was then heated on a water bath until the volume has been reduced by half. A yellow precipitate formed after standing at room temperature for 1 h and this was washed with ethanol. Yellow prisms were deposited from its acetonitrile solution within a week. Yield: 78%. *M*.pt: 112–113 °C. Anal. Found (calc'd for C_17_H_19_N_3_S_2_): C, 62.32 (61.97); H, 5.59 (5.81); N 12.80 (12.75).

### S3. Refinement

Carbon-bound H-atoms were placed in calculated positions (C—H = 0.95 to 0.99 Å) and were included in the refinement in the riding model approximation with *U*_iso_(H) = 1.2–1.5*U*_eq_(C). The N—H atom was refined with N—H = 0.88±0.01 Å, and with *U*_iso_(H) = 1.2*U*_eq_(N).

### Crystal data

**Table:**

C_17_H_19_N_3_S_2_	*F*(000) = 348
*M**_r_* = 329.47	*D*_x_ = 1.313 Mg m^−^^3^
Monoclinic, *P**c*	Cu *K*α radiation, λ = 1.5418 Å
*a* = 9.0073 (2) Å	Cell parameters from 12240 reflections
*b* = 12.3856 (2) Å	θ = 4–71°
*c* = 7.5553 (1) Å	µ = 2.88 mm^−^^1^
β = 98.620 (2)°	*T* = 100 K
*V* = 833.35 (3) Å^3^	Prism, yellow
*Z* = 2	0.31 × 0.22 × 0.18 mm

### Data collection

**Table:**

Agilent Xcalibur, Eos, Gemini diffractometer	3195 independent reflections
Radiation source: Enhance (Cu) X-ray Source	3191 reflections with *I* > 2σ(*I*)
Graphite monochromator	*R*_int_ = 0.021
Detector resolution: 16.1952 pixels mm^-1^	θ_max_ = 71.5°, θ_min_ = 3.6°
ω scans	*h* = −11→11
Absorption correction: multi-scan (*CrysAlis PRO*; Agilent, 2011)	*k* = −15→15
*T*_min_ = 0.43, *T*_max_ = 0.60	*l* = −9→9
16118 measured reflections	

### Refinement

**Table:**

Refinement on *F*^2^	Hydrogen site location: mixed
Least-squares matrix: full	H-atom parameters constrained
*R*[*F*^2^ > 2σ(*F*^2^)] = 0.030	*w* = 1/[σ^2^(*F*_o_^2^) + (0.0663*P*)^2^ + 0.1105*P*] where *P* = (*F*_o_^2^ + 2*F*_c_^2^)/3
*wR*(*F*^2^) = 0.081	(Δ/σ)_max_ = 0.001
*S* = 1.05	Δρ_max_ = 0.28 e Å^−^^3^
3195 reflections	Δρ_min_ = −0.30 e Å^−^^3^
205 parameters	Absolute structure: Flack *x* determined using 1558 quotients [(*I*^+^)-(*I*^-^)]/[(*I*^+^)+(*I*^-^)] (Parsons *et al.*, 2013).
3 restraints	Absolute structure parameter: −0.011 (13)

### Special details

**Table:**

Geometry. All e.s.d.'s (except the e.s.d. in the dihedral angle between two l.s. planes) are estimated using the full covariance matrix. The cell e.s.d.'s are taken into account individually in the estimation of e.s.d.'s in distances, angles and torsion angles; correlations between e.s.d.'s in cell parameters are only used when they are defined by crystal symmetry. An approximate (isotropic) treatment of cell e.s.d.'s is used for estimating e.s.d.'s involving l.s. planes.

### Fractional atomic coordinates and isotropic or equivalent isotropic displacement parameters (Å^2^)

**Table:**

	*x*	*y*	*z*	*U*_iso_*/*U*_eq_	
S1	−0.01004 (7)	0.74703 (4)	0.22309 (8)	0.01770 (17)	
S2	0.20197 (7)	0.59121 (5)	0.07598 (9)	0.02369 (18)	
N1	0.2382 (2)	0.80053 (17)	0.1071 (3)	0.0165 (4)	
H1N	0.324 (2)	0.788 (3)	0.069 (4)	0.020*	
N2	0.1881 (2)	0.90223 (17)	0.1402 (3)	0.0151 (4)	
N3	0.4835 (2)	0.88995 (18)	0.0262 (3)	0.0171 (4)	
C1	0.1524 (3)	0.7141 (2)	0.1305 (3)	0.0164 (5)	
C2	−0.0843 (3)	0.6138 (2)	0.2616 (4)	0.0227 (6)	
H2A	−0.1317	0.5817	0.1469	0.027*	
H2B	−0.0025	0.5655	0.3160	0.027*	
C3	−0.1993 (3)	0.6274 (2)	0.3865 (4)	0.0187 (5)	
C4	−0.1541 (3)	0.6423 (2)	0.5698 (4)	0.0211 (5)	
H4	−0.0502	0.6481	0.6153	0.025*	
C5	−0.2590 (3)	0.6487 (2)	0.6864 (4)	0.0232 (5)	
H5	−0.2259	0.6587	0.8107	0.028*	
C6	−0.4129 (3)	0.6409 (2)	0.6239 (4)	0.0224 (6)	
C6'	−0.5270 (4)	0.6430 (2)	0.7516 (4)	0.0309 (7)	
H6'1	−0.5477	0.7180	0.7812	0.046*	
H6'2	−0.4872	0.6038	0.8612	0.046*	
H6'3	−0.6200	0.6085	0.6950	0.046*	
C7	−0.4574 (3)	0.6281 (2)	0.4406 (4)	0.0239 (6)	
H7	−0.5613	0.6238	0.3946	0.029*	
C8	−0.3525 (3)	0.6216 (2)	0.3238 (4)	0.0221 (5)	
H8	−0.3857	0.6130	0.1992	0.027*	
C9'	0.1975 (3)	1.0925 (2)	0.1434 (4)	0.0174 (5)	
H9'1	0.1047	1.0801	0.1937	0.026*	
H9'2	0.1743	1.1322	0.0305	0.026*	
H9'3	0.2675	1.1348	0.2282	0.026*	
C9	0.2679 (3)	0.9857 (2)	0.1097 (3)	0.0149 (5)	
C10	0.4164 (3)	0.9860 (2)	0.0464 (3)	0.0148 (5)	
C11	0.6190 (3)	0.8899 (2)	−0.0256 (3)	0.0174 (5)	
H11	0.6657	0.8221	−0.0378	0.021*	
C12'	0.8495 (3)	0.9734 (2)	−0.1194 (3)	0.0198 (5)	
H12A	0.8776	1.0426	−0.1679	0.030*	
H12B	0.8470	0.9174	−0.2115	0.030*	
H12C	0.9233	0.9538	−0.0156	0.030*	
C12	0.6969 (3)	0.9832 (2)	−0.0632 (3)	0.0164 (5)	
C13	0.6260 (3)	1.0811 (2)	−0.0445 (3)	0.0174 (5)	
H13	0.6734	1.1468	−0.0692	0.021*	
C14	0.4852 (3)	1.0830 (2)	0.0107 (3)	0.0166 (5)	
H14	0.4363	1.1498	0.0239	0.020*	

### Atomic displacement parameters (Å^2^)

**Table:**

	*U*^11^	*U*^22^	*U*^33^	*U*^12^	*U*^13^	*U*^23^
S1	0.0175 (3)	0.0118 (3)	0.0255 (3)	−0.0019 (2)	0.0087 (2)	−0.0006 (2)
S2	0.0239 (3)	0.0140 (3)	0.0359 (4)	−0.0007 (3)	0.0131 (3)	−0.0039 (3)
N1	0.0152 (10)	0.0128 (10)	0.0220 (10)	−0.0006 (7)	0.0043 (8)	0.0006 (8)
N2	0.0138 (10)	0.0142 (11)	0.0165 (9)	−0.0007 (7)	0.0001 (8)	0.0002 (7)
N3	0.0147 (9)	0.0160 (10)	0.0198 (10)	−0.0023 (8)	0.0008 (8)	−0.0003 (8)
C1	0.0144 (11)	0.0196 (13)	0.0154 (11)	0.0002 (10)	0.0029 (9)	0.0003 (10)
C2	0.0276 (14)	0.0112 (12)	0.0326 (15)	−0.0066 (10)	0.0151 (11)	−0.0013 (10)
C3	0.0215 (12)	0.0098 (11)	0.0266 (13)	−0.0010 (10)	0.0096 (10)	0.0011 (9)
C4	0.0215 (12)	0.0131 (12)	0.0285 (13)	0.0016 (10)	0.0031 (10)	0.0021 (10)
C5	0.0324 (14)	0.0160 (13)	0.0219 (12)	0.0013 (10)	0.0061 (10)	0.0016 (10)
C6	0.0276 (14)	0.0090 (12)	0.0342 (14)	−0.0017 (10)	0.0162 (11)	0.0007 (10)
C6'	0.0382 (16)	0.0168 (13)	0.0434 (17)	−0.0032 (12)	0.0246 (13)	−0.0037 (12)
C7	0.0196 (13)	0.0149 (13)	0.0376 (15)	−0.0032 (10)	0.0057 (11)	−0.0035 (11)
C8	0.0252 (13)	0.0157 (13)	0.0256 (13)	−0.0042 (10)	0.0043 (10)	−0.0033 (10)
C9'	0.0166 (12)	0.0156 (13)	0.0196 (11)	0.0005 (8)	0.0012 (9)	−0.0007 (9)
C9	0.0147 (11)	0.0175 (12)	0.0115 (10)	−0.0010 (9)	−0.0019 (8)	0.0000 (8)
C10	0.0150 (11)	0.0167 (12)	0.0115 (10)	−0.0015 (9)	−0.0021 (8)	−0.0002 (9)
C11	0.0148 (11)	0.0171 (12)	0.0200 (12)	−0.0009 (9)	0.0018 (9)	−0.0001 (9)
C12'	0.0145 (11)	0.0229 (13)	0.0218 (12)	−0.0027 (9)	0.0023 (10)	0.0025 (10)
C12	0.0138 (12)	0.0217 (13)	0.0125 (10)	−0.0031 (9)	−0.0022 (9)	0.0002 (9)
C13	0.0171 (11)	0.0176 (12)	0.0166 (11)	−0.0040 (9)	−0.0005 (9)	0.0022 (9)
C14	0.0172 (11)	0.0155 (12)	0.0160 (12)	0.0002 (9)	−0.0008 (9)	−0.0001 (9)

### Geometric parameters (Å, º)

**Table:**

S1—C1	1.762 (3)	C6'—H6'2	0.9800
S1—C2	1.820 (3)	C6'—H6'3	0.9800
S2—C1	1.655 (3)	C7—C8	1.389 (4)
N1—C1	1.348 (3)	C7—H7	0.9500
N1—N2	1.374 (3)	C8—H8	0.9500
N1—H1N	0.872 (14)	C9'—C9	1.505 (3)
N2—C9	1.299 (3)	C9'—H9'1	0.9800
N3—C11	1.336 (3)	C9'—H9'2	0.9800
N3—C10	1.353 (3)	C9'—H9'3	0.9800
C2—C3	1.512 (4)	C9—C10	1.486 (3)
C2—H2A	0.9900	C10—C14	1.397 (4)
C2—H2B	0.9900	C11—C12	1.403 (4)
C3—C8	1.391 (4)	C11—H11	0.9500
C3—C4	1.396 (4)	C12'—C12	1.503 (3)
C4—C5	1.387 (4)	C12'—H12A	0.9800
C4—H4	0.9500	C12'—H12B	0.9800
C5—C6	1.399 (4)	C12'—H12C	0.9800
C5—H5	0.9500	C12—C13	1.388 (4)
C6—C7	1.392 (4)	C13—C14	1.394 (4)
C6—C6'	1.511 (3)	C13—H13	0.9500
C6'—H6'1	0.9800	C14—H14	0.9500
			
C1—S1—C2	101.58 (12)	C6—C7—H7	119.4
C1—N1—N2	119.6 (2)	C7—C8—C3	121.0 (3)
C1—N1—H1N	117 (3)	C7—C8—H8	119.5
N2—N1—H1N	123 (3)	C3—C8—H8	119.5
C9—N2—N1	119.5 (2)	C9—C9'—H9'1	109.5
C11—N3—C10	118.5 (2)	C9—C9'—H9'2	109.5
N1—C1—S2	121.07 (18)	H9'1—C9'—H9'2	109.5
N1—C1—S1	113.26 (19)	C9—C9'—H9'3	109.5
S2—C1—S1	125.66 (16)	H9'1—C9'—H9'3	109.5
C3—C2—S1	107.59 (18)	H9'2—C9'—H9'3	109.5
C3—C2—H2A	110.2	N2—C9—C10	127.3 (2)
S1—C2—H2A	110.2	N2—C9—C9'	114.3 (2)
C3—C2—H2B	110.2	C10—C9—C9'	118.4 (2)
S1—C2—H2B	110.2	N3—C10—C14	121.0 (2)
H2A—C2—H2B	108.5	N3—C10—C9	118.3 (2)
C8—C3—C4	118.2 (2)	C14—C10—C9	120.7 (2)
C8—C3—C2	121.2 (2)	N3—C11—C12	124.5 (2)
C4—C3—C2	120.6 (2)	N3—C11—H11	117.8
C5—C4—C3	120.8 (2)	C12—C11—H11	117.8
C5—C4—H4	119.6	C12—C12'—H12A	109.5
C3—C4—H4	119.6	C12—C12'—H12B	109.5
C4—C5—C6	121.1 (2)	H12A—C12'—H12B	109.5
C4—C5—H5	119.4	C12—C12'—H12C	109.5
C6—C5—H5	119.4	H12A—C12'—H12C	109.5
C7—C6—C5	117.8 (2)	H12B—C12'—H12C	109.5
C7—C6—C6'	121.0 (3)	C13—C12—C11	116.6 (2)
C5—C6—C6'	121.2 (3)	C13—C12—C12'	123.6 (2)
C6—C6'—H6'1	109.5	C11—C12—C12'	119.8 (2)
C6—C6'—H6'2	109.5	C12—C13—C14	119.9 (2)
H6'1—C6'—H6'2	109.5	C12—C13—H13	120.1
C6—C6'—H6'3	109.5	C14—C13—H13	120.1
H6'1—C6'—H6'3	109.5	C13—C14—C10	119.6 (2)
H6'2—C6'—H6'3	109.5	C13—C14—H14	120.2
C8—C7—C6	121.1 (2)	C10—C14—H14	120.2
C8—C7—H7	119.4		
			
C1—N1—N2—C9	−176.9 (2)	C2—C3—C8—C7	176.4 (3)
N2—N1—C1—S2	174.81 (17)	N1—N2—C9—C10	−2.4 (4)
N2—N1—C1—S1	−5.6 (3)	N1—N2—C9—C9'	177.39 (19)
C2—S1—C1—N1	−173.23 (18)	C11—N3—C10—C14	1.3 (3)
C2—S1—C1—S2	6.4 (2)	C11—N3—C10—C9	−178.1 (2)
C1—S1—C2—C3	164.66 (18)	N2—C9—C10—N3	−4.0 (4)
S1—C2—C3—C8	104.6 (2)	C9'—C9—C10—N3	176.2 (2)
S1—C2—C3—C4	−77.7 (3)	N2—C9—C10—C14	176.6 (2)
C8—C3—C4—C5	1.4 (4)	C9'—C9—C10—C14	−3.1 (3)
C2—C3—C4—C5	−176.4 (2)	C10—N3—C11—C12	−0.9 (4)
C3—C4—C5—C6	−0.2 (4)	N3—C11—C12—C13	−0.1 (4)
C4—C5—C6—C7	−1.0 (4)	N3—C11—C12—C12'	179.6 (2)
C4—C5—C6—C6'	177.3 (3)	C11—C12—C13—C14	0.5 (3)
C5—C6—C7—C8	1.1 (4)	C12'—C12—C13—C14	−179.1 (2)
C6'—C6—C7—C8	−177.3 (3)	C12—C13—C14—C10	−0.1 (3)
C6—C7—C8—C3	0.1 (4)	N3—C10—C14—C13	−0.9 (3)
C4—C3—C8—C7	−1.3 (4)	C9—C10—C14—C13	178.5 (2)

### Hydrogen-bond geometry (Å, º)

Cg1 is the centroid of the C3–C8 ring.

**Table:**

*D*—H···*A*	*D*—H	H···*A*	*D*···*A*	*D*—H···*A*
N1—H1*N*···N3	0.88 (2)	1.98 (3)	2.624 (3)	130 (3)
C12′—H12*C*···N2^i^	0.98	2.58	3.483 (3)	154
C13—H13···*Cg*1^ii^	0.95	2.76	3.582 (3)	145

Symmetry codes: (i) *x*+1, *y*, *z*; (ii) *x*+1, −*y*+2, *z*−1/2.
