# Supplementary figures and images for: Crystal structure of (Z)-4-methylbenzyl 3-[1-(5-methylpyridin-2-yl)ethylidene]dithiocarbazate
Source: Acta Crystallogr E Crystallogr Commun. 2015 Dec 19;71(Pt 12):o1071–2. doi: 10.1107/S205698901502407X (PMC4719984; doi:10.1107/S205698901502407X)

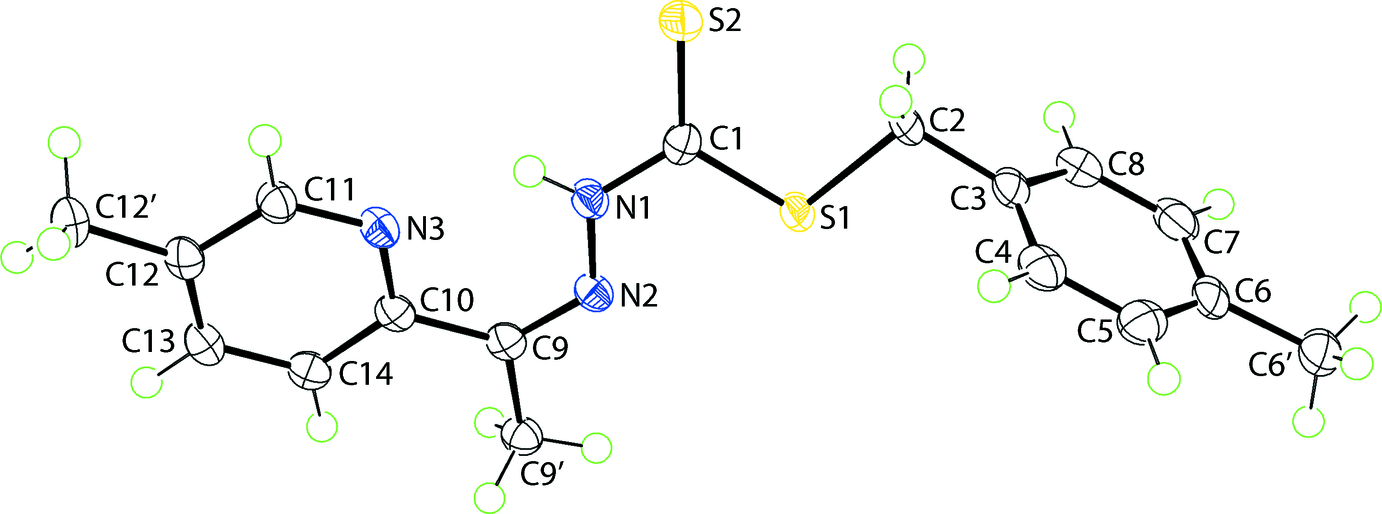

Supplement: Supplementary file 3 [file e-71-o1071-fig1.tif]

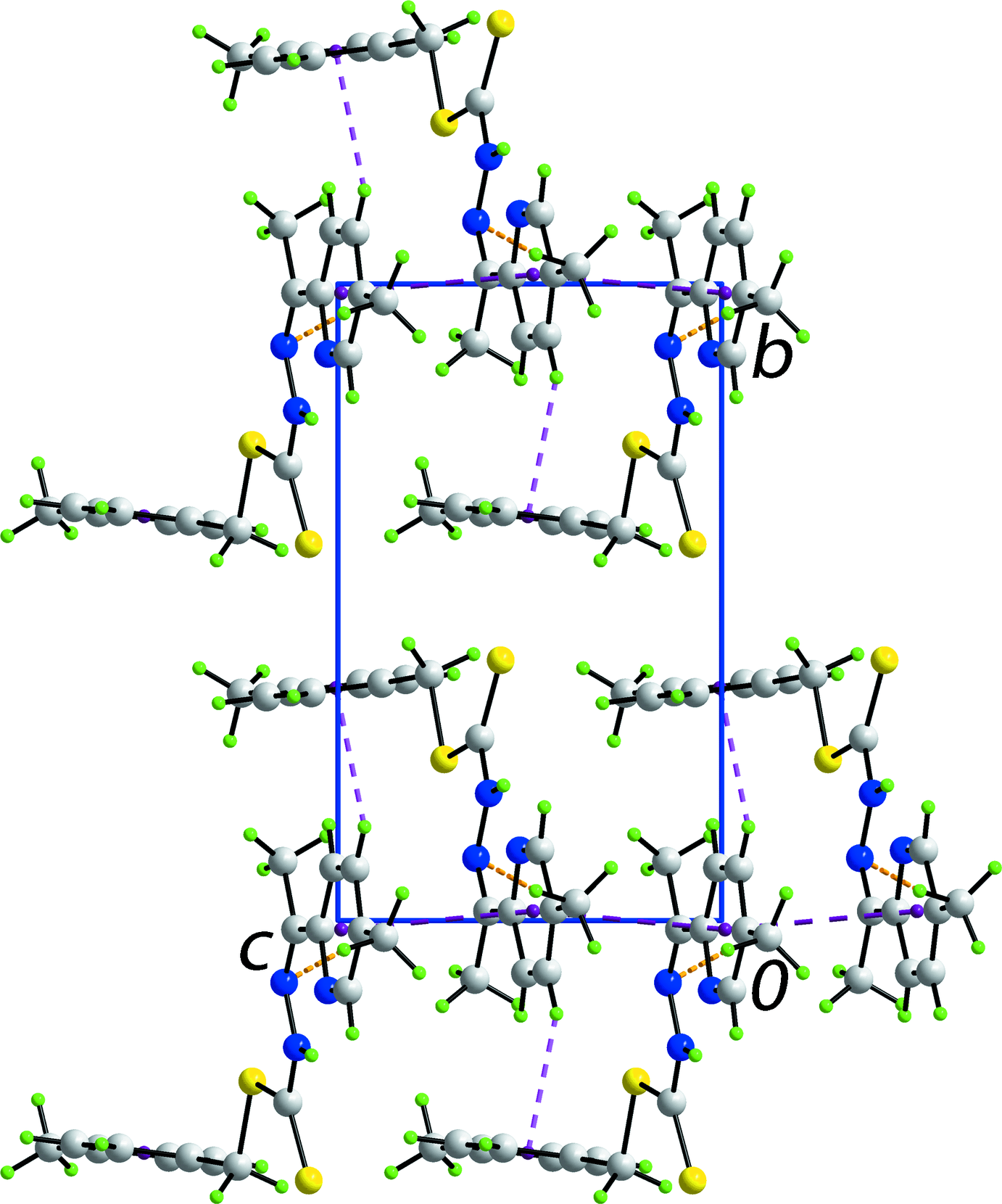

Supplement: Supplementary file 4 [file e-71-o1071-fig2.tif]
